# Germ cell depletion from mammalian ovary: possible involvement of apoptosis and autophagy

**DOI:** 10.1186/s12929-018-0438-0

**Published:** 2018-04-23

**Authors:** Pramod K. Yadav, Meenakshi Tiwari, Anumegha Gupta, Alka Sharma, Shilpa Prasad, Ashutosh N. Pandey, Shail K. Chaube

**Affiliations:** Cell Physiology Laboratory, Department of Zoology, Institute of Science, Banaras Hindu University, Varanasi, 221005 UP India

**Keywords:** Germ cell loss, Apoptosis, Autophagy, Mammalian ovary

## Abstract

Mammalian ovary contains millions of germ cells during embryonic life but only few of them are culminated into oocytes that achieve meiotic competency just prior to ovulation. The majority of germ cells are depleted from ovary through several pathways. Follicular atresia is one of the major events that eliminate germ cells from ovary by engaging apoptotic as well as non-apoptotic pathways of programmed cell death. Apoptosis is characterized by several morphological changes that include cell shrinkage, nuclear condensation, membrane blebbing and cytoplasmic fragmentation by both mitochondria- as well as death receptor-mediated pathways in encircling granulosa cells and oocyte. Although necroapoptosis have been implicated in germ cell depletion, autophagy seems to play an active role in the life and death decisions of ovarian follicles. Autophagy is morphologically characterized by intracellular reorganization of membranes and increased number of autophagic vesicles that engulf bulk cytoplasm as well as organelles. Autophagy begins with the encapsulation of cytoplasmic constituents in a membrane sac known as autophagosomes. The autophagic vesicles are then destroyed by the lysosomal enzymes such as hydrolases that results in follicular atresia. It seems that apoptosis as well as autophagy could play active roles in germ cells depletion from ovary. Hence, it is important to prevent these two pathways in order to retain the germ cells in ovary of several mammalian species that are either threatened or at the verge of extinction. The involvement of apoptosis and autophagy in germ cell depletion from mammalian ovary is reviewed and possible pathways have been proposed.

## Background

Mammalian ovary contains millions of germ cells during fetal life and few of them are culminated into oogonia [1, 2]. These oogonia enter into meiosis and get arrested at diplotene stage of first meiotic prophase [2, 3]. Oogonia are converted into primary oocytes during folliculogenesis in the ovary. The ovarian follicles contain diplotene arrested primary oocytes that remain arrested at this stage from birth to puberty [3–5]. The diplotene arrest is the longest period of oocyte meiosis in mammals [4]. Meiotic resumption from diplotene arrest is triggered by pituitary gonadotropins surge during puberty [6]. Oocyte progresses through metaphase-I (M-I) to metaphase-II (M-II) stage by extruding first polar body (PB-I) at the time of ovulation in several mammalian species [6–8]. After ovulation, oocyte is converted into egg and posses PB-I that indicate M-II arrest. Conversion of germ cell in oocyte may last from several months to years and by that time majority of germ cells are eliminated from the cohort of ovary probably by apoptosis and autophagy [3, 9]. Hence prevention of these two pathways would offer the availability of large number of germ cells in the cohort of ovary during reproductive lifespan of several mammalian species.

Ovary is a dynamic organ that rapidly and effectively eliminates excessive and defective germ cells to ensure the ovulation of viable, competent and good quality egg for fertilization and early embryonic development [3, 7, 8, 10, 11]. Therefore, ovary is able to ovulate less than 1% of high quality eggs, while majority of germ cells (> 99%) are eliminated from cohort of ovary during entire reproductive life [3, 8, 11, 12]. As the aging occurs, poor quality eggs are ovulated that declines fertility in several mammalian species [13]. Due to larger size (80–120 μm), mammalian oocytes are more susceptible towards reactive oxygen species (ROS) induced cell death that reduces germ cell’s number and oocyte quality in the ovary [5, 8, 11]. Although recent studies suggest the presence of oocyte like stem cell that opens a new source of oocyte in adult ovary, the quality of oocytes and their fate remains controversial [5, 8, 11].

The germ cells are eliminated from ovary by apoptotic as well as non apoptotic-mediated pathways of programmed cell death (PCD) [3, 14, 15]. Studies suggest that apoptosis as well as autophagy could be involved in the elimination of germ cell from ovary [3, 16, 17]. Apoptosis in oocyte has been reported at all the stages of oogenesis and even after ovulation [6, 16–18]. Oocyte apoptosis is morphologically characterized by cell shrinkage, nuclear condensation, membrane blebbing and formation of apoptotic bodies [3, 18, 19].

The autophagy is identified by the presence of cytoplasmic vesicles, which engulf cytoplasmic organelles [18, 20–24]. The autophagy starts by enclosing cytoplasmic constituents in a membrane sac called autophagosomes [18, 22, 24, 25]. The lysosomal hydrolases generally destroy autophagic vesicles and their contents in a cell [22, 23]. Indeed, apoptosis together with autophagy are involved in the elimination of majority of germ cells, therefore it is important to prevent these two pathways in order to protect germ cell depletion from ovary. Studies from our laboratory suggest that the use of enzymatic as well as non–enzymatic antioxidant, reduction of ROS level in the oocyte and inhibition of caspases activity prevent oocyte apoptosis through mitochondria-mediated pathway under in vivo as well as in vitro culture conditions [19, 26–30]. Thus, the prevention of germ cell depletion would provide an abundant source of oocytes in the ovary during reproductive life for live stock production in farm animals and enhance the reproductive potential in species that are at the verge of extinction [8]. This article review and update the information on the role of apoptosis and autophagy in germ cell depletion from mammalian ovary.

## Follicular atresia

In mammals, few number of follicles complete folliculogenesis and rupture to release competent oocytes [31–34]. Majority of follicles undergo atresia during folliculogenesis [35]. The PCD in encircling granulosa cells as well as oocyte may result in the collapse of entire follicle. This notion is supported by the observations that autophagy induces granulosa cell death in the follicle of human ovary [36–39]. Studies suggest that apoptosis is not only responsible for follicular atresia; autophagy is also involved in granulosa cell death during follicular atresia in goose and quail ovary [1]. A large number of germ cells, oogonia and oocytes are eliminated from the ovary via atresia. A different pattern of follicular atresia is observed during various follicular phases in mammalian ovary. In primordial, primary and small pre–antral follicle, oocyte apoptosis triggers follicular atresia. On the other hand, in late pre–antral, antral and pre–ovulatory follicles, granulosa cell death induces follicular atresia [22, 40, 41].

## Germ cell depletion via apoptosis

The involvement of apoptosis in germ cell depletion from mammalian ovary has recently been reviewed and possible pathways are proposed [3]. These include mitochondria-mediated (intrinsic) or death receptors-mediated (extrinsic) and some of them link these two pathways to eliminate germ cells from ovary [3, 17, 42]. Factors that induce generation of ROS follow mitochondria-mediated pathway to eliminate oocytes from ovary [3, 43–45]. The death receptors-mediated pathway is triggered by Fas ligand and procaspase-8 events. The caspase-8 activates caspase-3 that finally disrupts the histoarchitecture of a cell resulting in oocyte apoptosis [3, 30, 46, 47].

Oxidative stress (OS) is one of the major factors that induce oocyte apoptosis in mammals [3, 19]. The increased level of OS can modulate Bax/Bcl2 ratio and thereby mitochondria membrane potential (MMP) [29, 48–50]. Changes in MMP allow cytochrome c release from endoplasmic reticulum in the cytoplasm [3, 28, 30, 51]. BH3 only protein acts as a key regulator of apoptosis within the ovary [3, 17]. It links between mitochondria- and death receptor-mediated pathways. A truncated BID (tBID) induces overexpression of Bax that modulates MMP and cytochrome c release. The cytochrome c binds to apoptotic protease factor 1 in the oocyte cytoplasm that activates caspase-9 in oocyte cytoplasm. The active caspase-9 triggers conversion of procaspase-3 to caspase-3 [3, 28, 49]. Once activated, caspase-3 cleaves structural and regulatory protein in oocytes and results in the appearance of morphological apoptotic feature in oocyte [3, 28, 29, 49, 52–54].

**Fig. 1:**
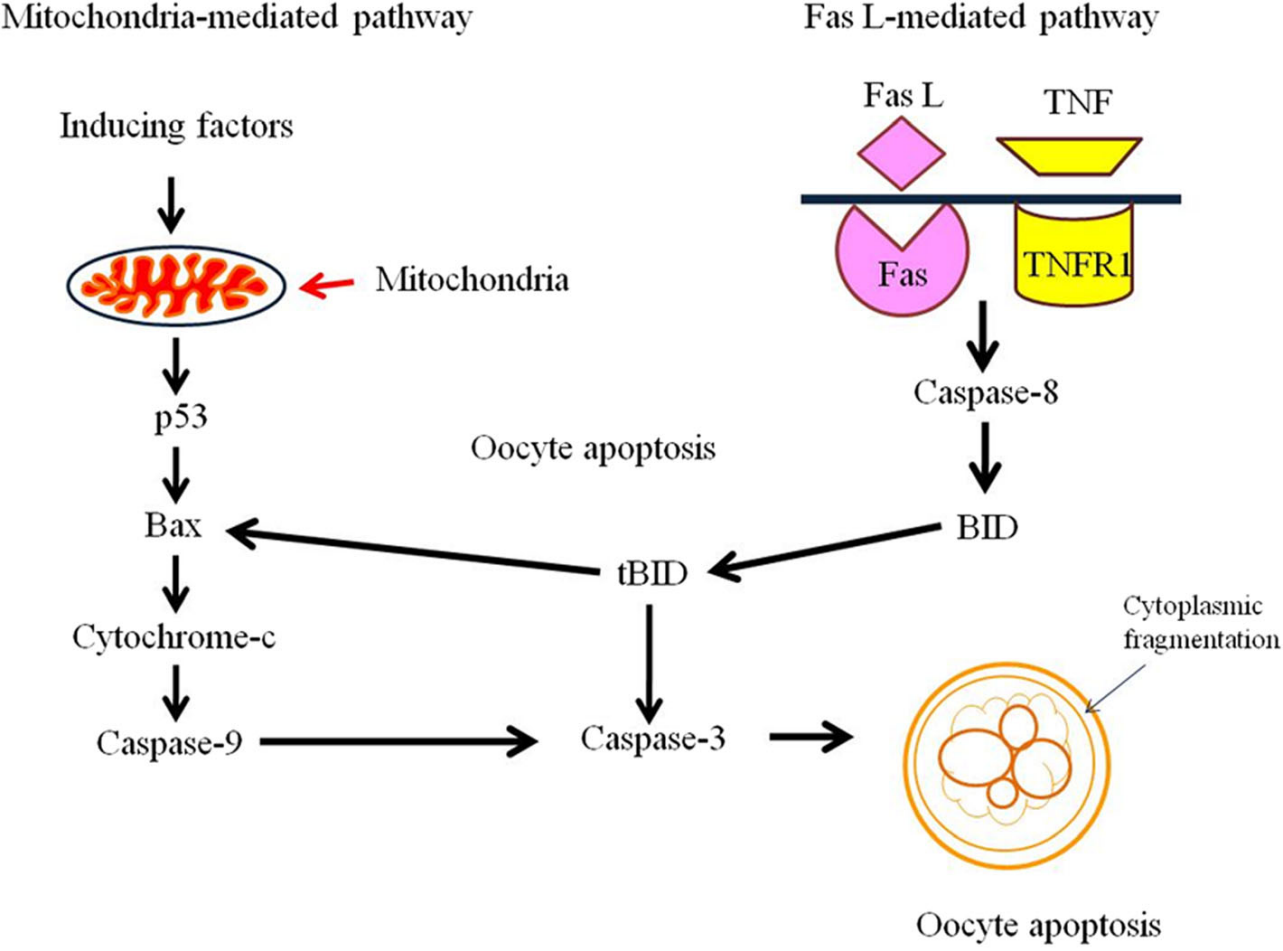
A schematic diagram showing the involvement of mitochondria- as well as death receptor-mediated pathways in oocyte apoptosis

The tumor necrosis factor receptor family such as Fas and tumor necrosis factor 1 (TNFR1) bind to their ligands (FasL and TNFα) to initiate death receptor-mediated apoptotic pathway [17, 30]. Studies suggest that the sustained reduced Thr-161 phosphorylated cyclin- dependent kinase 1 (Cdk1) and cyclin B1 levels destabilize maturation promoting factor (MPF) in rat oocyte [55, 56]. The increased FasL results in receptor trimerization and recruitment of Fas-associated death domain (FADD) containing protein [30, 57]. The FADD converts procaspase-8 into caspase-8. Once activated, caspase-8 converts BID into tBID on one hand and activates caspase-3 on the other hand to induce oocyte apoptosis (Fig. 1) [3, 46, 52, 58].

## Germ cell depletion via autophagy

The ‘autophagy’, is derived from the greek meaning ‘eating of self’. For the first time, the term autophagy was coined by Christian de Duve [59]. Autophagy is a complex process that involves degradation of abnormal proteins and organelles by autophagosome [60, 61]. The autophagosomes are double layered vesicles that act as intracellular cargo for the delivery of abnormal proteins and subcellular organelles to lysosomes for proteolytic degradation [20, 23, 24, 62–64]. Autophagy has step by step processing of abnormal proteins and surplus organelles through sequestration transport to lysosomes, degradation and utilization of degraded products. However, each step may have different functions [65].

The involvement of macroautophagy, microautophagy and chaperone-mediated autophagy in wide variety of cells and tissues has already been reviewed [66]. In macroautophagy, fusion of double membrane autophagosomes and lysosomes are involved, while in microautophagy a direct uptake of material by the lysosome similar to pinocytosis has been reported [66]. On the other hand, in chaperone-mediated autophagy, import of protein in lysosome and its interaction with protein chaperone takes place [20, 23, 64]. Autophagy is of great importance for the maintenance of cellular homeostasis under natural conditions. It is induced by nutrient starvation [67], amino acid deprivation [68, 69], hypoxia [70], oxidative stress [71], pathogen infection [72] and in damaged organelles [73, 74] such as endoplasmic reticulum [75].

Previously it was believed that autophagy is a survival mechanism under nutritional stress by degrading cytoplasmic material to generate energy for starving cell [20, 61, 76, 77]. By doing so, autophagy eliminates damaged and dysfunctional organelles, misfolded proteins and foreign particles such as microorganism to protect cellular demise [78–80]. Studies suggest that autophagy functions as a cytoprotective mechanism in the beginning, if the damage is extensive or in the absence of apoptosis, autophagy may induce cell death [81–83]. However, recent studies suggest that the autophagy has physiological as well as pathophysiological roles in starvation adaptation, organelle clearance, development, elimination of microorganisms, cell death and suppression etc. [65].

A growing body of evidences suggest that autophagy induces apoptosis by triggering the accumulation of autophagosomes in granulosa as well as luteal cells [36–39, 84–86]. This is supported by the observation that the activation of Akt and mTOR inhibit granulosa cell autophagy during folliculogenesis, while down regulation of Akt and mTOR induce granulosa cell autophagy and thereby follicular atresia [86]. The Akt inhibitor decreases follicle stimulating hormone (FSH)-mediated increase of mTOR activity and increased autophagy suggesting that Akt controls mTOR activity as well as autophagy in rat granulosa cells [86]. Based on these studies, we propose that the autophagy may directly be involved in follicular atresia and germ cell elimination from mammalian ovary. A possibility exist that the autophagy directly or indirectly via apoptosis plays a major role during massive granulosa cell death during follicular atresia. The molecular pathways that induce autophagy in granulosa cells during folliculogenesis and atresia remains ill understood. Based on existing studies, we propose that granulosa cell death either by apoptosis or autophagy or both may deprive follicular oocytes from nutrient growth factors, survival factors and cell cycle proteins required for the oocyte during final stages of oogenesis making oocyte susceptible towards apoptosis or autophagy or both.

The adult rat ovary shows the sign of both apoptotic as well as autophagic cell death [22]. More than 40% oocytes showed biochemical and morphological features of apoptosis and autophagy, suggesting the elimination of germ cell through a new form of PCD [22]. The combined roles of apoptosis as well autophagy in the elimination of oocytes have been studied using immature rat [83]. Studies suggest that the inhibition of autophagy increases apoptosis, while inhibition of apoptosis resulted in autophagy [83, 87]. In some cases, both apoptosis as well as autophagy are reported simultaneously [87]. Hence, apoptosis and autophagy functions together in the elimination of germ cells from pre pubertal rat ovary [87]. It has been observed that oocyte expresses one or more than one markers of autophagy suggesting that the autophagy plays an important role during germ cell elimination from rat ovary [87].

The autophagy consists of vesicle nucleation, elongation, maturation, fusion with lysosome and finally degradation [23, 60, 65, 74, 88]. The nutrient deprivation, hypoxia and damaged organelles in the oocytes may generate stress and thereby ROS. The accumulation of ROS and inhibition of antioxidant system results in OS that initiates the autophagy in wide variety of cells [25, 60, 65, 74, 89, 90]. The AMP-activated protein kinase (AMPK) is an energy sensor kinase initiates the cascade of autophagy by acting on the ULK complex and mammalian target of rapamycin complex (mTORC1) [25, 60, 65, 74, 89, 90]. The AMPK binding not only removes mTORC1 from ULK1/2 complex but also phosphorylates ULK1/2 complex. The phosphorylated ULK1/2 triggers phosphorylation of other units of the same complex in order to activate the whole complex [60, 64, 65, 73, 74]. The activated ULK1/2 complex recruits endoplasmic reticulum, mitochondria and plasma membrane for the formation of phagophore [25, 60, 74, 91].

For phagophore formation, Class III phosphatidylinositol 3-kinase (Class III PI3K) complex is required that consist of factors like Beclin1, Vps34 and Vps15 [60, 64, 74, 92]. The Ambra1/Atg14L and UV resistance-associated gene protein (UVRAG)/Bif-1 are also the subunits of class III PI3K complex [60, 62, 74, 93]. Beclin1 is one of the important units of Class III PI3K and closely related to anti-apoptotic protein Bcl-2. Under normal conditions, Beclin1 binds to Bcl-2 and causes inhibition of autophagy but under stressed condition Beclin1 dissociates from Bcl-2 [25, 60, 64, 74, 93].

Phosphorylation of Bcl-2 causes the separation of Beclin1 from Bcl-2. The dephosphorylated Beclin1 interacts with Vps34 and generates the phosphatidylinositol-3-phosphate, responsible for phagophore generation [60, 62, 74, 94]. Other proteins like UVRAG and Atg14L interact with Beclin1 and responsible for phagophore maturation [60, 64, 73, 74]. The Atg12-Atg5 conjugation and microtubule-associated protein 1 light chain 3 (LC3) processing are important for elongation and maturation of phagophore. The E1-like enzyme Atg7 and E2-like enzyme Atg10 and other factor Atg16L help in the formation of Atg12-Atg5-Atg16L oligomer. These oligomers localize on the outer membrane of elongating phagophore [25, 60, 64, 74, 91].

**Fig. 2:**
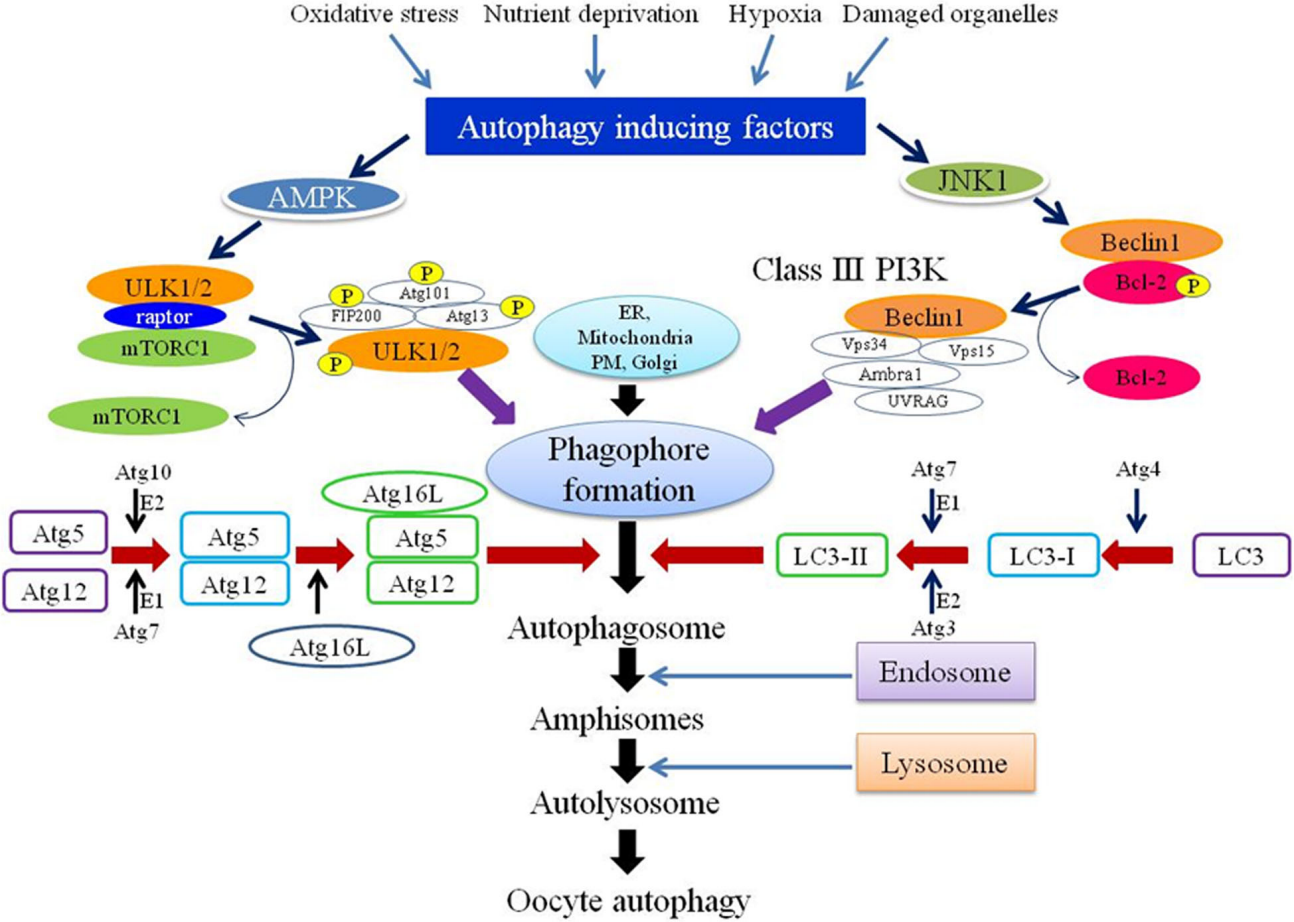
A schematic diagram showing possible pathways involved in oocyte autophagy

The Atg12-Atg5-Atg16L complex is required for the recruitment of LC3-II on outer membrane of phagophore. Further, the LC3 is also involved in the elongation and maturation of phagophore. The Atg4 converts LC3 into LC3-I and it is further converted into LC3-II with the help of E1 like enzyme Atg7 and E2 like enzyme Atg3 [60, 64, 74, 95]. The LC3-II fuses with phagophore membrane and form autophagosome [25, 60, 64, 74, 95, 96]. The fusion of endosome with autophagosome results in the formation of amphisomes. Further, the fusion of amphisomes with lysosome forms autolysosome [60, 64, 74, 97]. The autolysosome finally induces autophagy and products released in cytosol are utilized by the cells in various metabolic pathways (Fig. 2) [60, 73, 74].

## Conclusions

Millions of germ cells are present in the cohort of ovary during fetal development but ovary losses majority of these germ cells before they start working. Apoptosis alone or in combination with autophagy could play major roles in germ cell depletion from mammalian ovary. The prevention of both apoptosis as well as autophagy during reproductive life of mammalian female would allow the availability of good quality of oocytes in the ovary in order to preserve ovarian reserve and thereby female fertility in mammalian ovary including human.

## Abbreviations

AMPK: AMP-activated protein kinase
FADD: Fas-associated death domain containing protein
LC3: microtubule-associated protein 1 light chain 3
MMP: Mitochondria membrane potential
mTORC1: mammalian target of rapamycin complex
OS: Oxidative stress
PCD: Programmed cell death
ROS: Reactive oxygen species
